# A Diversity, Equity, and Inclusion (DEI) Framework for Addressing Gender Disparities in the Nursing Profession

**DOI:** 10.7759/cureus.91265

**Published:** 2025-08-29

**Authors:** Khina Sharma, Vikas Chaudhary, Jyoti Kathwal, Anoop Sharma, Shivani Bhardwaj

**Affiliations:** 1 College of Nursing, All India Institute of Medical Sciences, Jodhpur, IND; 2 College of Nursing, All India Institute of Medical Sciences, Bilaspur, IND; 3 Neurology, All India Institute of Medical Sciences, Bilaspur, IND

**Keywords:** dei in nursing, gender diversity, gender equity in nursing, male nurses, men in nursing

## Abstract

Nursing remains a female-dominated profession, with men constituting a small but growing minority. Despite ongoing diversity, equity, and inclusion (DEI) initiatives, male nurses continue to face stereotypes, biases, and systemic barriers.

A literature review was conducted using peer-reviewed studies and systematic reviews on male nurses' experiences. Key themes explored included male nurses, gender-based stereotypes, workplace integration, and professional equity. Policies affecting male nurses, such as gender-based reservations and role assignments, were also analyzed.

Findings indicate that while efforts to increase male representation in nursing exist, societal perceptions continue to discourage their entry. Male nurses report exclusionary practices, such as gender-based reservation in recruitment and gendered role assignments and biases that affect career progression. While some experience advantages in leadership, others face discrimination in patient care settings, particularly in specialties like obstetrics and pediatrics.

Despite DEI initiatives, true gender inclusivity in nursing remains a challenge. Institutions must promote gender-neutral recruitment, equitable leadership pathways, and mentorship programs for male nurses. Addressing implicit biases and dismantling stereotypes will foster a more inclusive nursing workforce. Ensuring fair treatment for all genders will strengthen the profession and improve patient care outcomes.

## Introduction and background

Diversity, equity, and inclusion (DEI) initiatives help create a positive workplace culture globally. It also plays a vital role in the nursing profession. DEI promotes fair representation, fosters mutual respect, and enhances the quality of patient care. Diversity ensures that nurses from various cultural, ethnic, and social backgrounds are represented, which in turn leads to improved patient care by integrating a range of perspectives and experiences. Equity focuses on fair treatment, ensuring that all nurses have equal access to opportunities, professional growth, and resources, regardless of their gender, race, or socioeconomic background. Inclusion fosters a sense of belonging, where every nurse feels respected, valued, and empowered to contribute to patient care [1]. A strong DEI framework in nursing not only improves teamwork and job satisfaction but also enhances competent care, leading to better health outcomes for diverse patient populations.

Nursing has long been considered a female-dominated profession, with men historically playing a significant role before a shift in perception occurred. In many parts of the world, the nursing workforce remains disproportionately female. According to the World Health Organization's State of the World's Nursing report, men make up only about 10% of the global nursing workforce [2]. However, historically, men were actively involved in nursing, particularly within monastic orders and military contexts, providing care for the sick and injured. The world's first nursing school, established in India around 250 BC, was exclusively for men. Male nurses also played crucial roles during the Crusades, staffed field hospitals in the Franco-Prussian War of 1870, and served on the front lines during World War I. However, despite having the same training as their female counterparts, they were often referred to as "orderlies" and received lower pay. The profession's gender shift largely stemmed from Florence Nightingale's influence, as she redefined nursing as a career primarily suited for women. Some scholars argue that her belief that "every woman is a nurse by nature" may have contributed to the ongoing underrepresentation of men in nursing [3].

Since the 1960s, efforts have been made to promote gender inclusivity in nursing, leading to a gradual increase in male participation. The National Council of State Boards of Nursing in the United States conducted a National Nursing Workforce Survey in 2020, revealing that men comprised 9.4% of registered nurses, compared to 9.1% in 2017, 8% in 2015, and 6.6% in 2013 [4]. While these figures indicate progress, men in nursing continue to face challenges and barriers. Over the last few decades, the number of men entering the nursing profession has seen a steady rise. According to the US Census Bureau, only 2.7% of registered nurses in 1970 were male. By 2011, that figure had increased to 9.6% [5]. 

Nurse shortage and male nurses 

Nurses frequently serve as the primary, and sometimes the sole, point of contact for patients within the healthcare system, making them vital contributors to addressing health-related challenges at every level [6]. Despite their critical role, a significant obstacle to achieving global health targets, including the Sustainable Development Goals (SDGs), is the persistent and growing shortage of nursing professionals. Projections suggest that by the next decade, the world could face a shortfall of approximately 13.5 million nurses [7]. The World Health Organization has estimated that to meet healthcare demands by 2030, an additional six million nurses will be required, representing a 20% increase over the current global workforce of 27.9 million. Additionally, with nearly 4.7 million nurses anticipated to retire during this period, substantial recruitment efforts will be necessary to maintain even the current staffing levels [6]. The impact of the COVID-19 pandemic may further exacerbate this crisis, with preliminary data indicating that up to 10% of the global nursing workforce may leave the profession [7]. To meet future demands, it is essential to encourage a larger and more diverse group of individuals to enter the nursing profession, regardless of gender.

Although the number of men entering nursing has increased in recent years, their representation remains limited in many regions and particularly low in certain specialties such as obstetric and maternal healthcare [8]. This gender imbalance may be shaped by various factors, including societal expectations, traditional gender roles, and public perceptions of nursing as a predominantly female profession. These perceptions often relate to issues such as professional status, compensation, and working conditions [9]. In some countries, a higher proportion of male nurses may reflect limited opportunities for women in other fields, while in others, the reverse may be true [6]. To address the global nursing shortage effectively, it is essential to adopt inclusive recruitment strategies that encourage greater gender diversity, particularly by supporting more men to enter the profession. As conversations around DEI evolve, the role of men in nursing warrants closer examination. Despite efforts to promote gender inclusivity, men still face challenges such as stereotypes, biases, and systemic barriers that hinder their full integration into the profession. This raises an important question: Are men truly included in DEI initiatives within nursing, or do they remain an overlooked minority? Addressing these issues is essential to fostering a more inclusive and equitable nursing workforce.

## Review

Diversity: representation of men in nursing

Diversity in nursing is often viewed through the lens of cultural and racial representation; however, gender diversity plays an equally important role in building an inclusive and well-rounded healthcare workforce. Although the number of men entering nursing is gradually increasing, they still represent a minority within the profession. This imbalance is largely shaped by longstanding societal perceptions that label nursing as a "woman's job", a belief reinforced by historical norms, educational narratives, and media portrayals. Nevertheless, gender should never define one's ability to provide compassionate and competent care. Encouragingly, in part decades, India is witnessing a shift. According to a World Health Organization report, at least 20.5% of nurses in India were male, a proportion that exceeds the global average [10]. While this marks progress, the representation of men in nursing remains significantly lower than that of women, indicating that more work is needed to promote gender balance in the field.

Research has shown that societal norms and gender biases often deter men from entering the nursing profession, contributing to their continued underrepresentation [8]. Clinical settings can further compound this issue by lacking inclusivity, which may hinder male nurses from fully engaging in their roles. Moreover, male nursing students and professionals frequently receive minimal support or even face discouragement from peers due to their minority status in the field [11]. A significant concern also arises around physical touch in patient care; male nurses may worry that their actions could be misinterpreted, potentially exposing them to allegations of inappropriate conduct [12].

Despite all efforts, men in nursing continue to experience discrimination, social stigma, and exclusion, particularly in fields like obstetrics and gynecology. These challenges contribute to high dropout rates among male nursing students and lower retention in the profession. Alarmingly, male nurses leave the profession at nearly twice the rate of their female counterparts (7.5% versus 4.1%) within four years of graduation. This is a concerning increase from 1992 data, which showed similar early-career departure rates between genders (2% male vs. 2.7% female). The conflict between traditionally masculine traits, such as strength, leadership, and assertiveness, and the emotional qualities often associated with nursing, like empathy and gentleness, can create internal conflicts for many male nurses [13].

Equity: do men receive fair opportunities?

Equity in nursing should ensure that all nurses, regardless of gender, have equal access to career growth, leadership roles, and professional development opportunities. However, the reality is more complex. While some argue that men in nursing may enjoy advantages in leadership and promotions due to gender biases favoring men in management roles, others highlight that the majority of male nurses face significant barriers in nursing education and clinical practice [14].

Historically, male nurses have experienced considerable exclusion and discrimination within the nursing profession, even in developed countries like the United States. For example, the American Nurses Association (ANA) did not permit male membership between 1898 and 1930. During the 19th and early 20th centuries, men were systematically excluded from formal nursing organizations. In 1901, when the US Army Nurse Corps was officially established, it was composed exclusively of women, despite the fact that men had previously served in similar roles as early as the 1850s. This gender-based exclusion persisted until 1955, when male nurses were finally allowed to serve in the Army Nurse Corps [15].

A similar pattern of discrimination has been observed in India, where men were historically appointed as nursing orderlies rather than recognized as professional nurses. Although they were allowed to enroll in diploma-level nursing programs, male candidates were excluded from degree-level courses and often overlooked in direct recruitment processes. These practices reflect a long-standing gender bias that has hindered the full inclusion and equity of men in the nursing profession [16].

Currently, a significant gender disparity exists at the undergraduate level in central government institutions in India, such as the All India Institute of Medical Sciences (AIIMS), where seats are not allocated for male candidates in nursing programs. This lack of opportunity poses a major hurdle for aspiring male nurses, often forcing them to pursue education in private institutions where tuition fees are considerably higher. In contrast, postgraduate nursing programs at AIIMS offer equal opportunities to both male and female candidates, reflecting a more balanced approach at the advanced level of education [17].

Moreover, India's 80:20 reservation policy in nursing recruitment within central institutes, where 80% of positions are reserved for female candidates and only 20% for male candidates, has further contributed to gender disparity within the profession. While the policy may aim to maintain traditional workforce composition, it is rooted in deep-seated stereotypes and societal prejudices, particularly the belief that nursing is inherently a feminine profession and that men are less suited for caregiving roles. Also, in military nursing services, male nurses are not permitted to serve as nurses in the Indian Army; they can only join as nursing orderlies. It is unfortunate that, although male nurses were historically trained for military service, they are now barred from entering this field. Such policies inadvertently restrict opportunities for men who wish to enter and advance in the profession. These practices reinforce gendered assumptions about caregiving and limit the career prospects of male nurses in India [18].

Inclusion: do men feel welcomed in nursing?

Inclusion in nursing entails creating an environment where individuals of all genders feel respected, valued, and supported in their professional roles. However, many male nurses continue to face challenges that question the inclusivity of the profession. Some of these challenges include exclusion from nursing textbooks, facing skepticism from patients, receiving stereotypical task assignments, experiencing feelings of isolation when working in predominantly female teams, and being frequently mistaken for doctors, etc. [14].

One of the major contributing factors to the sense of exclusion is the gender bias evident in nursing textbooks. The historical account of nursing in the majority of textbooks begins with the entry of women into the profession around 300 AD, completely overlooking the contributions of male caregivers in ancient Greece and Rome nearly a thousand years earlier. This omission reflects a broader trend within nursing education, where the role of men is often ignored. Furthermore, nursing literature frequently employs female pronouns and examples, reinforcing the stereotype of nursing as a female-only profession and failing to represent the true gender diversity within the field [16].

These educational biases extend into clinical practice, where gender-based assumptions often influence task assignments. Male nurses are commonly perceived as more suitable for physically demanding roles, such as those in trauma care, orthopedic units, or surgical wards. In contrast, specialties like obstetrics and gynecology, pediatrics, and community health are often seen as more appropriate for female nurses. However, this belief is contradicted by the presence of male physicians who successfully practice in these same fields, highlighting that professional roles in healthcare should be determined by competence, not gender. Supporting this, a qualitative study conducted in Tanzania revealed that male nurses were often delegated tasks involving physical strength or technical expertise, reinforcing stereotypical role divisions and limiting their exposure to more holistic aspects of patient care. Moreover, it has been observed that men are more often encouraged or assigned to specialties characterized by "high-tech, low-touch" care, where emotional and physical caregiving are less emphasized, further reflecting underlying gender biases in clinical practice [19].

Persistent social stereotypes continue to challenge gender equity, with many perceiving male nurses as less compassionate or questioning their career choices. Common misconceptions include viewing nursing as inherently feminine or making unwarranted assumptions about male nurses' sexuality, which can lead to workplace discrimination and professional dissatisfaction. A systematic review demonstrated how such biases negatively impact male nurses' professional identity and sense of belonging [20]. However, emerging research offers a more nuanced perspective. For example, a study conducted in India revealed that 67.5% of participants held neutral views, 58.7% rejected the stereotype of nursing being feminine, and 89.5% reported no gender preference for their caregivers [21]. These findings provide an evidence-based foundation for challenging gender biases and reevaluating restrictive policies such as "female-only" rules.

Earlier research exploring the workplace experiences of male nurses revealed that they often face challenges in building comfortable professional relationships with female colleagues and may feel isolated within teams [22]. Additionally, male nurses are frequently subjected to differing expectations compared to female nurses [8]. Variations in job roles, work settings, and access to professional opportunities based on gender can foster discriminatory practices. Many male nurses have reported encountering gender-based discrimination in their workplaces. One report found that 44% of male nurses experienced gender-based discrimination during their careers, while 31% expressed feelings of social isolation [23]. Similarly, a study reported that men entering the profession faced multiple barriers, including prevailing stereotypes, inadequate recruitment strategies targeting men, and a lack of visible male role models in both academia and media [24]. Supporting this, a survey of 498 male nurses found that 73% encountered gender-based stereotypes, 59% perceived nursing as a female profession, and 53% believed that alternative careers were more socially acceptable for men [8].

Research further suggests that higher attrition rates among male nurses are linked to multiple workplace challenges such as role ambiguity, a lack of professional acceptance, inadequate salaries, limited professional recognition, dissatisfaction with the work environment, and restricted opportunities for career advancement [25]. Importantly, this attrition is not limited to job turnover but often results in complete withdrawal from the profession. Several studies have also emphasized that male nurses face obstacles in establishing long-term careers, particularly due to the underrepresentation of men in leadership and supervisory roles, which are still predominantly occupied by women [26]. These pressures not only affect professional identity but also significantly impact job satisfaction and retention. For instance, in the United States, 46% of male nurses reported having considered leaving the profession. In China, the intention to resign among male nurses ranged from 11.1% to 85.7%, driven by burnout, low self-efficacy, and negative workplace environments. Furthermore, male nursing graduates often experience higher unemployment rates, 7.5% compared to 4.1% for female graduates [13].

**Figure 1:**
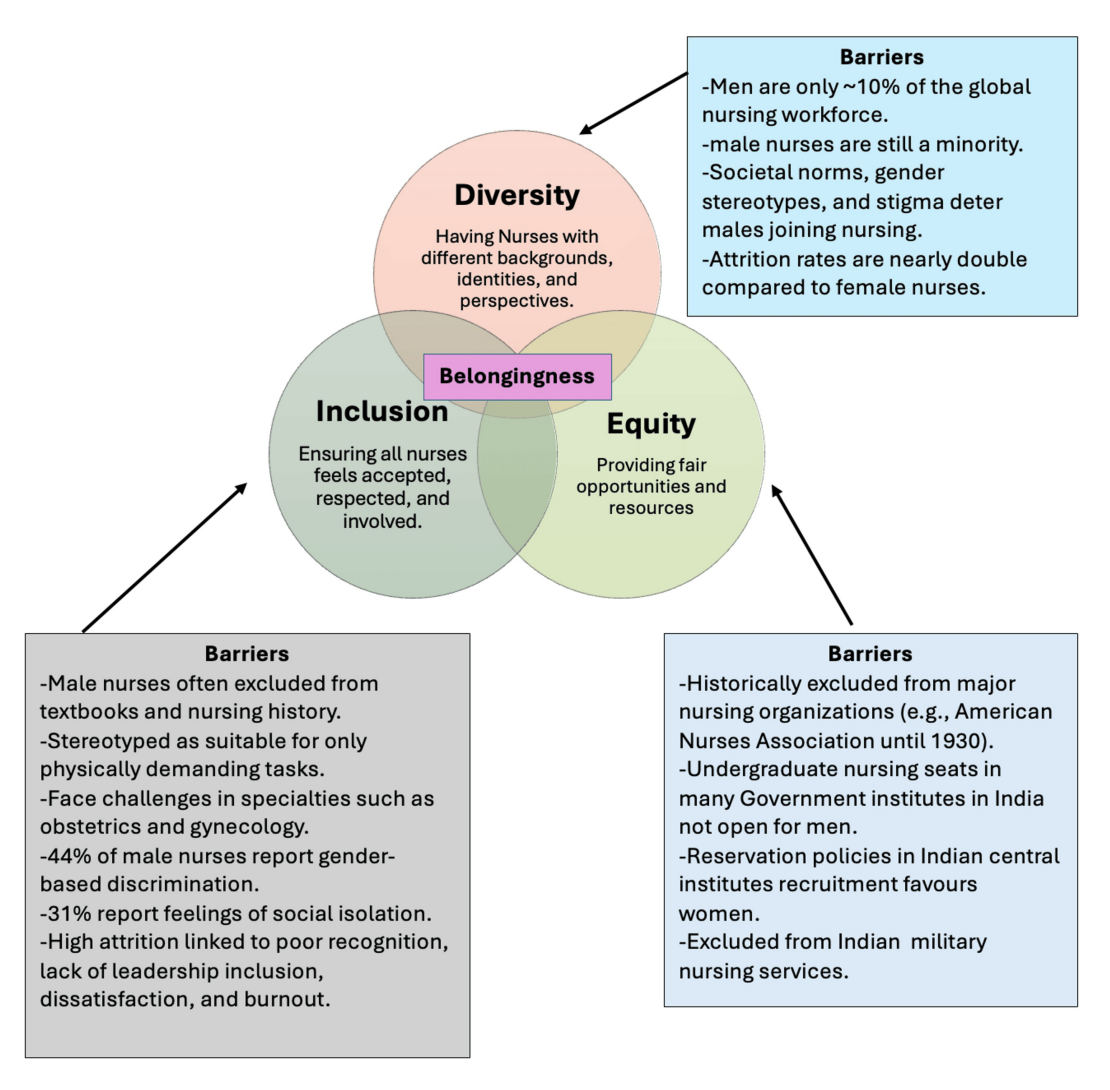
Diversity, equity, and inclusion framework addressing gender disparities and its barriers in the nursing profession

Figure 1 highlights the need for a more inclusive and gender-equitable approach in nursing education and workplace culture. Promoting male representation, addressing biases, and fostering supportive environments are crucial steps toward ensuring that nursing becomes a truly inclusive profession for all.

Ways to improve DEI in terms of gender in nursing 

Encouraging more men to pursue nursing is not about replacing one group with another; rather, it is about building a diverse, equitable, and inclusive profession enriched by a wide range of experiences and perspectives. Achieving true gender equity in nursing requires a multifaceted approach, one that not only increases male representation but also fosters an environment in which all professionals can thrive. Some of the key strategies to advance gender-focused DEI in nursing are as follows:

Equal Opportunity for All to Enter the Profession

To create a truly inclusive nursing profession, equal access to education and career entry must be ensured for individuals of all genders. Historically, nursing has been viewed as a female-dominated field, which often discourages men from applying due to entrenched social norms. Institutions must actively dismantle such barriers by eliminating gendered language from admissions criteria, application forms, and promotional materials. Faculty and admissions committees should undergo training to identify and overcome biases that may inadvertently disadvantage male applicants. Inclusive outreach should highlight that compassion, clinical acumen, and leadership are not gender-specific qualities, thereby attracting diverse candidates into the field [8].

Targeted Scholarships and Awareness Campaigns

Financial constraints and social stigma often prevent men from pursuing nursing as a profession. Targeted scholarships for deserving and financially needy male nursing students can help reduce the financial burden and serve as a motivation to enter the field. In parallel, awareness campaigns should be strategically designed to challenge stereotypes that associate nursing solely with women. These campaigns can include success stories of male nurses, interviews, social media content, and community engagement activities that emphasize nursing as a science-based, people-centered, and highly rewarding profession for everyone. Tailoring these messages to reach young men in schools and colleges can be especially effective [10,25]. 

Gender-Neutral Recruitment Strategies

Traditional recruitment strategies often unconsciously favor female applicants through language and imagery that reinforce outdated gender norms. 80:20 gender-based recruitment quota in central government hospitals is such an example. Gender-neutral recruitment involves using inclusive language that emphasizes professional skills, such as clinical judgment, empathy, and decision-making, instead of gender-coded terms. Visuals and advertisements should reflect both men and women in varied roles, settings, and levels of responsibility. This shift in messaging plays a critical role in reshaping the public perception of nursing [14].

Public Outreach Programs

Introducing nursing as a viable and honorable career choice during early education can significantly influence future decisions. Outreach programs targeting middle and high school students, especially young boys, can demystify misconceptions about the profession. These initiatives can include school visits by male nurses, informational sessions for parents, interactive workshops, and shadowing opportunities. One such example is the "20x20: Choose Nursing" campaign, launched by the American Association for Men in Nursing (AAMN), aimed to increase male representation in nursing to 20% by 2020 through outreach, education, and mentoring [27].

Mentorship Programs for Male Nurses

Mentorship is a powerful tool in supporting professional growth, especially for individuals from underrepresented groups. For male nurses, mentorship offers guidance through academic challenges, clinical rotations, and workplace dynamics. Having role models who share similar experiences can help alleviate the feelings of isolation often reported by male nursing students and early-career professionals. Structured mentorship programs can also promote retention, boost confidence, and prepare mentees for advanced roles such as clinical specialists, educators, and nurse leaders. Peer support groups and alumni networks can further strengthen these initiatives [28].

Inclusive Organizational Policies

Policies within healthcare institutions should explicitly reflect principles of gender equity and inclusion. These policies must go beyond formal statements and be operationalized in practice. Examples include fair task distribution based on skills rather than physical assumptions, ensuring male nurses have equal access to work in maternal-child health settings, and maintaining respectful communication in the workplace. Gender-inclusive language in official documents, diversity training, and a zero-tolerance approach to discrimination must be part of institutional culture. Leaders must monitor policy implementation to ensure consistency and accountability [25].

Addressing Implicit Biases

Unconscious biases can shape attitudes and behaviors toward male nurses, influencing assignments, evaluations, and workplace relationships. Male nurses are often stereotyped as less nurturing or unsuitable for roles in neonatal or pediatric units. These assumptions can hinder learning opportunities and career progression. To counter this, academic and clinical settings should offer bias-awareness workshops, scenario-based learning, and regular feedback mechanisms. Institutions must reinforce a culture where competence, not gender, determines opportunities and responsibilities. Encouraging open conversations about gender perceptions can lead to more inclusive professional dynamics [23].

Valuing Diverse Perspectives

A diverse nursing workforce offers varied viewpoints that enrich patient care, team collaboration, and clinical decision-making. Male nurses contribute unique experiences and approaches that can enhance the profession's adaptability and responsiveness. Their perspectives can also influence leadership styles, communication techniques, and conflict resolution strategies within healthcare teams. Celebrating these differences not only fosters inclusivity but also improves organizational effectiveness. Institutions should move beyond mere representation and actively recognize the value that men bring to nursing through awards, research grants, and speaking platforms [29].

Professional Development Opportunities

Career advancement opportunities should be equally accessible to all nurses, regardless of gender. Often, men may not be encouraged to pursue leadership or specialization tracks due to societal assumptions or lack of visibility in those roles. Institutions should implement transparent criteria for promotions, provide leadership development programs, and actively encourage male nurses to participate in professional forums, conferences, and academic advancement. Tracking participation by gender can also help identify gaps and guide targeted interventions [8].

Media Portrayal

Mainstream media continues to depict male nurses in a limited and often comical light, reinforcing stereotypes that undermine their professional identity. These portrayals influence not only public perception but also the self-image of aspiring male nurses. Nursing organizations should collaborate with media producers to ensure accurate and respectful representations of male nurses. Documentaries, news features, and promotional films should highlight the competencies and contributions of male nurses across diverse settings such as emergency care, mental health, administration, and research. Balanced media representation is a key component in reshaping societal attitudes and boosting male engagement in the profession [30].

## Conclusions

While DEI are foundational principles in modern nursing, true gender inclusivity remains an ongoing challenge. Men in nursing continue to navigate barriers that affect their full participation and acceptance in the field. To achieve genuine DEI in nursing, the profession must actively dismantle stereotypes, implement supportive policies, and foster a culture where all nurses, regardless of gender, feel valued and empowered to contribute meaningfully to patient care and professional growth. By embracing gender diversity in its truest form, nursing can evolve into a profession that is truly inclusive for all.​
